# Elevated *TNFRSF4* gene expression is a predictor of poor prognosis in non-M3 acute myeloid leukemia

**DOI:** 10.1186/s12935-020-01213-y

**Published:** 2020-05-04

**Authors:** Siyu Gu, Jie Zi, Qi Han, Chunhua Song, Zheng Ge

**Affiliations:** 1 Department of Hematology, Zhongda Hospital, School of Medicine, Southeast University, Institute of Hematology Southeast University, No. 87, Dingjiaqiao, Gulou District, Nanjing, 210009 Jiangsu China; 2 Hershey Medical Center, Pennsylvania State University Medical College, Hershey, PA17033 USA

**Keywords:** *TNFRSF4*, AML, TCGA, Bioinformatics

## Abstract

**Background:**

We used bioinformatic tools to dichotomize 157 non-M3 AML patients from the TCGA dataset based on the presence or absence of *TP53* mutations, and screened out a key gene related to *TP53* mutation for future analysis.

**Methods:**

DEGs were analyzed by R package “DESeq2” and then run GSEA, GO enrichment, KEGG pathway and PPI network. Hub genes were selected out according to MCC. Log-rank (Mantel–Cox) test was used for survival analysis. Mann–Whitney U’s nonparametric t test and Fisher’s exact test was used for continuous and categorical variables respectively. *p* value< 0.05 was considered to be statistical significance.

**Results:**

*TNFRSF4* was final screened out as a key gene. Besides *TP53* mutation (*p *= 0.0118), high *TNFRSF4* was also associated with *FLT3* mutation (*p *= 0.0102) and *NPM1* mutation (*p *= 0.0024). Elevated *TNFRSF4* was significantly related with intermediate (*p *= 0.0004) and poor (*p *= 0.0011) risk stratification as well as relapse statute (*p *= 0.0099). Patients with elevated *TNFRSF4* expression had significantly shorter overall survival (median survival: 2.35 months vs. 21 months, *p *< 0.0001). Based on our clinical center data, *TNFRSF4* expression was significantly higher in non-M3 AML patients than HDs (*p *= 0.0377) and MDS patients (EB-1, 2; *p *= 0.0017).

**Conclusions:**

Elevated *TNFRSF4* expression was associated with *TP53*, *FLT3 and NPM1* mutation as well as poor clinical outcome. *TNFRSF4* expression was significantly higher in non-M3 AML patients than HDs and MDS (EB-1, 2) patients. *TNFRSF4* is need for future functional and mechanistic studies to investigate the role in non-M3 AML.

## Background

Acute myeloid leukemia (AML) is a heterogenous and hematologic malignant disease which is characterized by infiltration of the bone marrow, blood, and other tissues by proliferative, clonal, abnormally differentiated, and occasionally poorly differentiated cells of the hematopoietic system [1]. Abnormal accumulated blasts replace the normal hematopoietic tissue and trigger out cytopenia [2]. With the advance of microarray and next-generation sequencing, recognition of the molecular heterogeneity of AML has enormously increased [3, 4]. However, current understanding of the molecular mechanisms underlying the development and progression of AML is limited, and early diagnosis remains difficult, which may lead to treatment delays. Therefore, the identification of key mechanisms regulating AML management and patient survival may contribute to the development of AML specific targeted therapies.

Since 1989, *TP53* has been identified as a tumor suppressor gene [5], which encodes tumor suppressor p53 protein regarded as “guardian of the genome” that plays an important role in maintaining genome stability under cellular stress, and participating in various processes of development, differentiation, aging, and disease [6, 7]. *TP53* mutations account for ~ 10% of de novo AML patients [8], 20–37% of secondary AML, therapy-relate AML patients [9] and 60% of complex karyotype patients. *TP53* mutations are also increasingly common appearance in relapsed or refractory AML cases which predicts poor clinical outcome [10, 11].

Tumor necrosis factor receptor superfamily member 4 (TNFRSF4), as known as OX40 or CD134 is expressed primarily on activated T cells [12]. TNFRSF4 can activate the NF-kappa-B pathway by mediating TRAF2 and TRAF5 [13]. The PI3K/PKB and NFAT pathway also have been identified as the downstream of TNFRSF4 [12, 14]. The most remarkable function of TNFRSF4 is to enhance division, proliferation, survival and cytokine production of T cells by activating the pathways described above. Series researches have investigated that TNFRSF4 as a therapeutic agent plays a significant role in immunotherapy of preclinical tumor models [15–17].

It has been found that *TP53* mutations promote the immunogenicity of breast cancer, and elevated *TNFRSF4* expression is also associated with *TP53* mutations [18]. On the other hand, TNFRSF4 expression in CD8-positive (CD8+) T cells and Tregs is significantly increased in relapsed AML patients compared with healthy donors (HDs) [19]. We analyzed the differentially expressed genes (DEGs) function or pathways between *TP53*-mutated and *TP53*-wildtype non-M3 AML based on the Cancer Genome Atlas (TCGA) transcriptome data [3]. *TNFRSF4* was finally screened out as a key gene associated with poor clinical outcome. In addition, based on our clinical center data, we validated *TNFRSF4* expression level of non-M3 AML patients was significantly higher than that of HDs and Myelodysplastic syndrome (MDS) Excess blasts (EB)-1,2 patients. Furthermore, the expression level was positive related with the percentage of bone marrow blasts after combined with MDS patient data.

## Methods

### Patient datasets

Complete clinical data and RNA sequence data of 157 newly diagnosis adult non-M3 AML patients obtained from TCGA dataset [3] downloaded from cBioPortal (https://www.cbioportal.org) including normalized Z-score data and median expression data [20]. Z-score indicates the number of standard deviations away from the mean of expression in the reference population. The subtypes which were classified according to the French–American–British (FAB) classification systems in which M3 subtype was removed from present research considering the unique attributes [21]. The risk group stratification was according to National Comprehensive Cancer Network (NCCN) guidelines. Patients included in the study were assessed for the most frequently found somatic mutations in AML, such as *FLT3*, *NPM1*, *IDH1/2*, and *TET*.

Approval of the code of ethics and consent to participation are not necessary because all data is public to identify and all datasets analyzed in this study were available from cBioportal.

### DEGs analysis

Patients were dichotomized based on the presence and absence of *TP53* mutation. DEGs were filtrated by R package “DESeq2” [22]. The screening condition was to satisfy both log2FoldChange(log2FC) > 1 or < − 1, and adjusted *p* value < 0.05. All genes were visualized using volcanic map plotted by R package “ggplot2” [23].

### KEGG, GO and GSEA analysis

The Kyoto Encyclopedia of Genes and Genomes (KEGG) pathway analysis and Gene Ontology (GO) enrichment analysis were performed using Database for Annotation, Visualization and Integrated Discovery (DAVID https://david.ncifcrf.gov) online tool [24]. False discovery rate (FDR) < 0.05 was considered to indicate a statistically significant difference.

All genes were performed Gene set enrichment analysis (GSEA) with a cut off nominal *p* value< 0.05 and FDR < 0.10. The reference gene set from the Molecular Signatures Database (MSigDB) of c6, the oncogenic signatures which were generated directly from microarray data from National Center for Biotechnology Information Gene Expression Omnibus or from internal unpublished profiling experiments involving perturbation of known cancer genes [25].

### Protein–protein interaction (PPI) network analysis

Proteins and their functional interactions networks of selected enrichment genes were acquired from the STRING database (https://string-db.org) [26]. Genes with minimum interaction score more than 0.4 were selected to visualize in Cytoscape which is an open source software project to integrate biomolecular interaction networks with high throughput expression data and other molecular states into a unified conceptual framework [27]. We utilized CytoHubba plug-ins for ranking nodes in a network by their network features with the Maximal Clique Centrality (MCC) methods. Wayne diagram was produced by webtool Bioinformatics & Evolutionary Genomics (http://bioinformatics.psb.ugent.be/webtools/Venn/) to overlap genes.

### Clinical patients and reverse transcribed quantitative PCR (RT-qPCR)

To analyze the mRNA expression of *TNFRSF4* in human bone marrow cells (BMCs), We collected non-M3 de novo AML patients, MDS (EB-1, 2) and HDs from our clinical center. The written informed consents were provided by all the patients in accordance with the Declaration of Helsinki before enrollment in the study.

The total RNA was isolated by Trizol (Invitrogen, USA), and was reverse transcribed into cDNA using the PrimeScript™ RT Master Mix (Perfect Real Time) (TaKaRa, Dalian, China). RT-qPCR was performed using TaKaRa SYBR Supermix (TaKaRa, Dalian, China) on a StepOne Plus analysis system (Applied Biosystems, Foster City, CA, USA). The amplification conditions were as follows: pre-denaturation (95 °C for 30 s), 40 cycles of denaturation (95 °C for 30 s), and annealing and extension (60 °C for 34 s). The primers were designed and synthesized with the following sequences:

- sense, 5′-ACAACGACGTGGTCAGCTCCAA-3′,
- antisense, 5′-CAGCGGCAGACTGTGTCCTGT-3′(*TNFRSF4*);
- sense, 5′-GTAACCCGTTGAACCCCATT-3′,
- antisense, 5′-CCATCCAATCGGTAGTAGCG-3′ (18 s RNA).

The relative expression levels of the target genes were calculated by the comparative Ct method presented as 2^−ΔCt^. The experiments were conducted in triplicate.

### Statistical analysis

All data were analyzed with the IBM SPSS statistics 26 and GraphPad Prism 8 software. Log-rank (Mantel–Cox) test was used to compare Overall survival (OS) and disease free survival (DFS) between patients with Z ≥ 0 (high) and Z < 0 (low) *TNFRSF4* expression. Additionally, Kaplan–Meier survival curves were generated for patients with Z ≥ 0 and Z < 0 *TNFRSF4* expression after stratification by FAB classification, risk stratification, age, cytogenetic status, transplant status, *TP53*, *FLT3*, *NPM1* and *RUNX1* mutation status. Mann–Whitney U’s nonparametric t test and Fisher’s exact test was used for continuous and categorical variables respectively. Spearman rank correlation was used to analyze the correlation between *TNFRSF4* and bone marrow blasts. *p* value< 0.05 was considered to be statistical significance, and all statistical methods were list in Additional file 1: Table S1.

## Results

### DEGs analysis

**Fig. 1:**
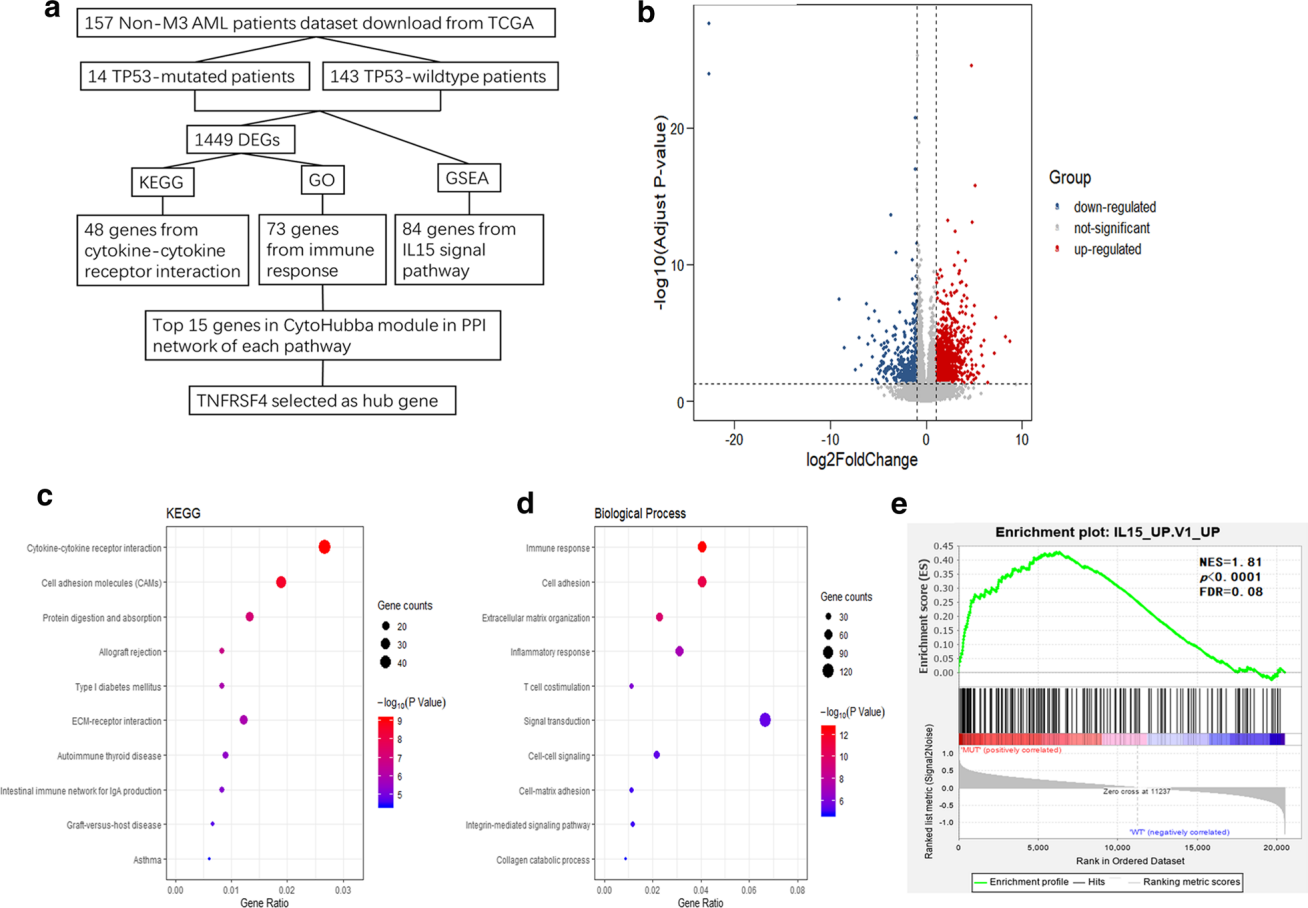
**a** The schematic view of the procedure of present study. **b** Volcanic maps of all genes. Red spot, the expression is up-regulated; Blue spot, the expression is down-regulated; Gray spot, no significantly dysregulated. **c**, **d** The bubble chart showed the top 10 pathways with significant difference. **c** The KEGG enrichment analysis. **d** The GO biological process enrichment analysis. **e** IL15 signaling pathway screened out from GSEA with the reference gene sets from the MSigDB of the oncogenic signatures

The present study was conducted as a multiple strategy to select hub genes correlated with *TP53* mutation from TCGA non-M3 AML patient dataset for further analysis (Fig. 1a). Patients were divided into *TP53* mutation and *TP53* wild type groups. 1449 DEGs were screened out by R package “DESeq2” and displayed in a volcanic map (Fig. 1b).

### KEGG and GO enrichment analysis

DEGs were conducted the KEGG as well as GO biological process enrichment analysis and the cytokine–cytokine receptor interaction pathway (*p *= 8.26E−10, FDR = 1.08E−06) and the immune response pathway (*p *= 2.53E−13, FDR = 4.68E−10) were screened out respectively (Fig. 1c, d).

### GSEA analysis

GSEA with the advantage of analyzing the genes obtained in the TCGA dataset instead of the DEGs. The IL15 signaling pathway was final screened out (NES = 1.81, *p *< 0.0001, FDR = 0.08, Fig. 1e). We next utilized the Wayne diagram and found 5 same genes, *TNFRSF4*, *TNFRSF9*, *CCL4*, *LIF* and *IL18RAP* by overlapping DEGs from cytokine–cytokine receptor interaction pathway, immune response pathway and the IL15 signaling pathway (Additional file 2: Figure S1A).

### PPI network

**Fig. 2:**
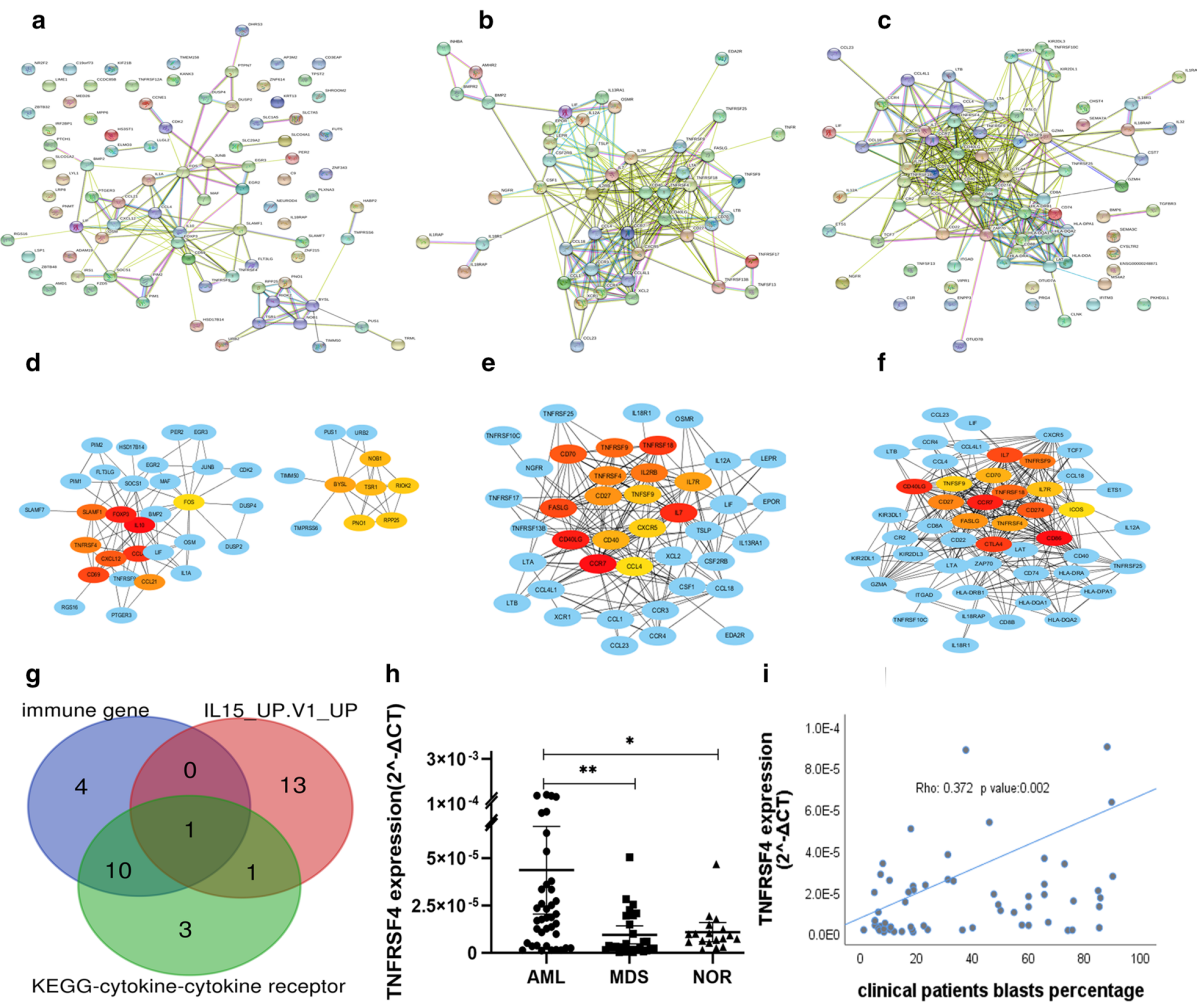
**a**–**c** The PPI analysis at STRING (https://string-db.org). **d**–**f** Cytoscape analysis candidate genes after PPI network analysis. **a**, **d** genes from IL15 signaling pathway; **b**, **e** genes from cytokine–cytokine receptor interaction pathway; **c**, **f** genes from immune response pathway. **g** Venn diagram showed the overlapping gene of candidate genes. **h** The *TNFRSF4* mRNA relative expression was calculated by the comparative Ct method presented as 2^−ΔCt^. *p < 0.05, **p < 0.01. **i** Spearman correlation coefficient analysis between bone marrow blasts percentage and *TNFRSF4* expression

In order to find the hub genes, we performed the PPI analysis of the DEGs from 3 most significant pathways aforementioned utilized the “String” website tool (Fig. 2a–c). Then we imported PPI networks into Cytoscape plug-ins to rank nodes and found out the candidate genes (Fig. 2d–f). We listed the 15 candidate genes of each pathways (Additional file 1: Table S2) by using CytoHubba plug-ins from Cytoscape software, and then screened out *TNFRSF4* by overlapping candidate genes from each pathway (Fig. 2g).

### *TNFRSF4* expression in bone marrow simples

We checked the TCGA normal and The Genotype-Tissue Expression AML datasets at GEPIA2 (http://gepia2.cancer-pku.cn) and found the *TNFRSF4* expression in AML is significantly higher than HDs (Additional file 2: Figure S1B). Subsequently, BM samples of 39 non-M3 AML, 29 MDS (EB-1, 2) patients and 18 HDs were collected from our clinical center, Southeast University affiliate Zhongda Hospital, from 1 February 2016 to 1 August 2019. RT-qPCR was performed to detect the *TNFRSF4* mRNA expression. The expression of *TNFRSF4* mRNA in AML patients was significantly higher compared with HDs (*p *= 0.0377) and MDS (EB-1, 2; *p *= 0.0017; Fig. 2h) patients respectively. There no statistically significant difference of *TNFRSF4* mRNA expression between HDs and MDS (EB-1, 2; *p *= 0.1243) patients.

Additionally, combined with MDS (EB-1, 2) patients blasts results, we found *TNFRSF4* expression level was positively related with bone marrow blasts percentage (Spearman’s Rho = 0.372, *p *= 0.002; Fig. 2i).

### *TNFRSF4* expression and clinical characters

*TNFRSF4* expression data and clinical data of 157 non-M3 patients were download from TCGA dataset. Histograms representing the distribution of *TNFRSF4* mRNA log2-transformed data and *TNFRSF4* scores are provided in Additional file 2: Figure S1C, D. The scatterplot of *TNFRSF4* log2-transformed mRNA expression against *TNFRSF4* Z score is shown in Additional file 2: Figure S1E (Pearson’s r = 0.8, *p *< 0.001). *TNFRSF4* mRNA levels were compared among patients classified according to the FAB and there was significant difference of *TNFRSF4* expression among the subgroups (Additional file 2: Figure S1F; *p *= 0.0055). We also analyzed *TNFRSF4* expression according to the NCCN AML classification based on their molecular and cytogenetic risk status into good, intermediate, and poor risk stratifications. *TNFRSF4* expression of good stratification patients was significantly lower than intermediate (*p *= 0.0004) and poor (*p *= 0.0011, Additional file 2: Figure S1G) stratification patients. Additionally, we used the Vizome data analysis tool [28], which contains data from the BEAT AML cohort, and examined the level of *TNFRSF4* expression of relapsed (N = 22) was significantly higher than de novo non-M3 AML (N = 214) samples (*p *= 0.0099, Additional file 2: Figure S1H).

We dichotomized the patients in the TCGA data set based on their *TNFRSF4* mRNA expression Z score (RNA Seq V2 RSEM) into high (Z score ≥ 0, N = 20) and low (Z score < 0, N = 137). Patients with high *TNFRSF4* expression had significantly higher age (median 66.5 vs. 57, *p *= 0.012) and were widely distributed in poor group (20% vs. 0%, *p *= 0.044) compared with the good group (Table 1).

**Table 1:** Clinical characteristics of non-M3 AML cohort in the TCGA data set with respect to *TNFRSF4* expression

	Total	*TNFRSF4* low (Z < 0)	*TNFRSF4* high (Z ≥ 0)	*p* value	
				*TNFRSF4* low vs. high	Fisher exact test
Sex, no. (%)					
Female	72	59 (81.9)	13 (18.1)		0.092
Male	85	78 (91.8)	7 (8.2)		
Age, years (range)				0.012	
Median	59	57	66.5		
Mean	56.0 ± 1.29	54.9 ± 1.38	63.5 ± 3.24		
WBC count ×109/L				0.528	
Median	22.2	22.2	25.95		
Mean	39.3 ± 3.76	37.0 ± 3.62	54.6 ± 15.93		
PB blasts, %				0.254	
Median	72	71	76.5		
Mean	68.4 ± 1.51	67.7 ± 1.62	72.7 ± 4.13		
BM blasts, %				0.06	
Median	42	39	58.5		
Mean	41.5 ± 2.58	39.5 ± 2.73	54.9 ± 7.32		
NCCN subtype, no.					Vs. favorable
Favorable	17	17 (100)	0 (0)		
Intermediate	92	82 (89.1)	10 (10.9)		0.169
Poor	45	36 (80)	9 (20)		0.044
FAB subtype, no.					
M0	16	13	3		
M1	44	36	8		
M2	38	33	5		
M4	34	32	2		
M5	18	17	1		
M6	2	2	0		
M7	3	2	1		

### *TNFRSF4* and mutations

**Fig. 3:**
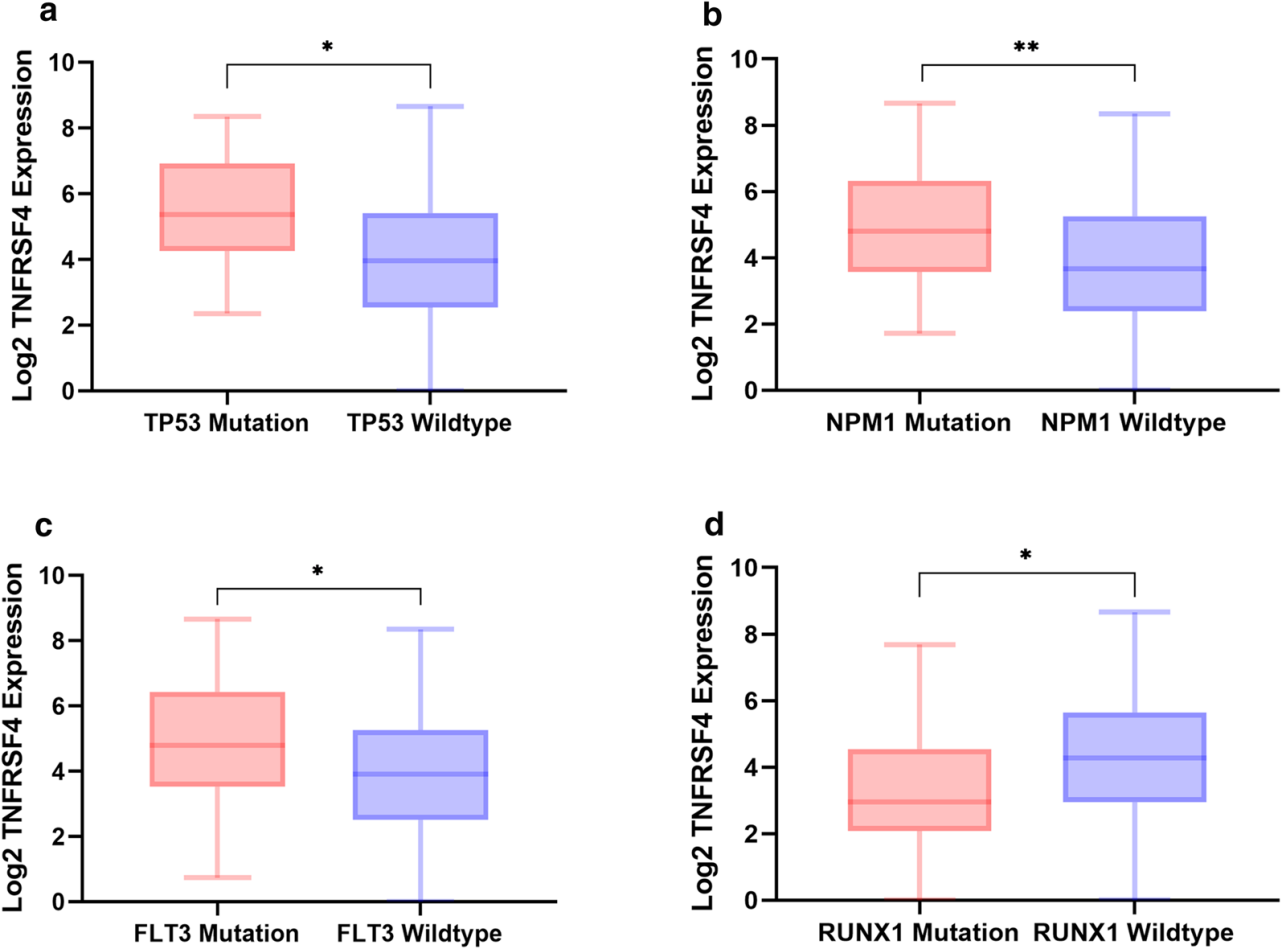
Association of *TNFRSF4* expression with patient mutational status. Relative *TNFRSF4* log2 mRNA expression in **a** patients with *TP53* mutations versus wild type; **b** patients with *NPM1* mutations versus wild type; **c** patients with *FLT3* mutations versus wild type; and **d** patients with *RUNX1* mutations versus wild type. *p < 0.05. **p < 0.01

*TNFRSF4* was screened out from non-M3 AML patients based on absence or presence of *TP53* mutation. We verified the expression of *TNFRSF4* was significantly higher in patients with *TP53* mutation (N = 15) than *TP53* wide type (N = 142, *p *= 0.0118, Fig. 3a). To understand other potential molecular genetic aberrations that may lead to or be associated with high *TNFRSF4*, we analyzed the expression with respect to the other mutational status of patients. *TNFRSF4* was significantly higher in patients with *NPM1* mutation (N = 49) than in patients with *NPM1* wild type (N = 108, *p* = 0.0024, Fig. 3b). *TNFRSF4* was also significantly higher in patients with *FLT3* mutation (ITD and point mutations) (N = 45) than in patients carrying *FLT3* wild type (N = 112, *p* = 0.0102, Fig. 3c). Additionally, *TNFRSF4* was significantly lower in the patients with *RUNX1* mutation (N = 28) than in patients with the wild-type *RUNX1* (N = 129, *p* = 0.0311, Fig. 3d). No significant association was observed between *TNFRSF4* expression and mutations in *DNMT3A*, *IDH1*, *IDH2*, *TET2*, *CEBPA*, *WT1*, and *NRAS*.

When we dichotomized patients according to *TNFRSF4* Z scores, we found a higher frequency of *TP53* mutations in *TNFRSF4* (Z ≥ 0) patients than in low *TNFRSF4* (Z < 0) patients (25% vs. 7.3%, Fisher exact, *p *= 0.026). No other association was found between *TNFRSF4* upregulation and mutations in *DNMT3A*, *IDH1*, *IDH2*, *TET2*, *NPM1*, *CEBPA*, *WT1*, and *NRAS* (Table 2).

**Table 2:** Association of *TNFRSF4* expression based on patient mutation status in the TCGA data set according to *TNFRSF4* expression

	Total	*TNFRSF4* low (Z < 0)	*TNFRSF4* high (Z ≥ 0)	*p* value	
				MUT vs. WT	Fischer exact test
*FLT3*, no. (%)				0.0102	0.111
MUT	45 (28.7)	36 (80.0)	9 (20.0)		
WT	112 (71.3)	101 (90.2)	11 (9.8)		
*IDH1*, no. (%)				0.6846	0.695
MUT	16 (10.2)	15 (93.7)	1 (6.3)		
WT	141 (89.8)	122 (86.5)	19 (13.5)		
*IDH2*, no. (%)				0.9227	0.699
MUT	17 (10.8)	16 (94.1)	1 (5.9)		
WT	140 (89.2)	121 (86.4)	19 (13.6)		
*RUNX1*, no. (%)				0.0311	0.759
MUT	28 (17.8)	24 (85.7)	4 (14.3)		
WT	129 (82.2)	113 (87.6)	16 (12.4)		
*TET2*, no. (%)				0.6979	0.103
MUT	15 (9.6)	11 (73.3)	4 (26.7)		
WT	142 (90.4)	126 (88.7)	16 (11.3)		
*NRAS*, no. (%)				0.4658	1
MUT	13 (8.3)	12 (92.3)	1 (7.7)		
WT	144 (91.7)	125 (86.8)	19 (13.2)		
*CEBPA*, no. (%)				0.7981	1
MUT	13 (8.3)	12 (92.3)	1 (7.7)		
WT	144 (91.7)	125 (86.8)	19 (13.2)		
*WT1*, no. (%)				0.736	0.363
MUT	10 (6.4)	10 (100.0)	0 (0.0)		
WT	147 (93.6)	127 (86.4)	20 (13.6)		
*DNMT3A,* no. (%)				0.6877	0.178
MUT	42 (26.8)	34 (81.0)	8 (19.0)		
WT	115 (73.2)	103 (89.6)	12 (10.4)		
*NPM1*, no. (%)				0.0024	0.439
MUT	49 (31.2)	41 (83.7)	8 (16.3)		
WT	108 (68.8)	96 (88.9)	12 (11.1)		
*TP53,* no. (%)				0.0118	0.026
MUT	15 (9.6)	10 (66.7)	5 (33.3)		
WT	142 (90.4)	127 (89.4)	15 (10.6)		

**Fig. 4:**
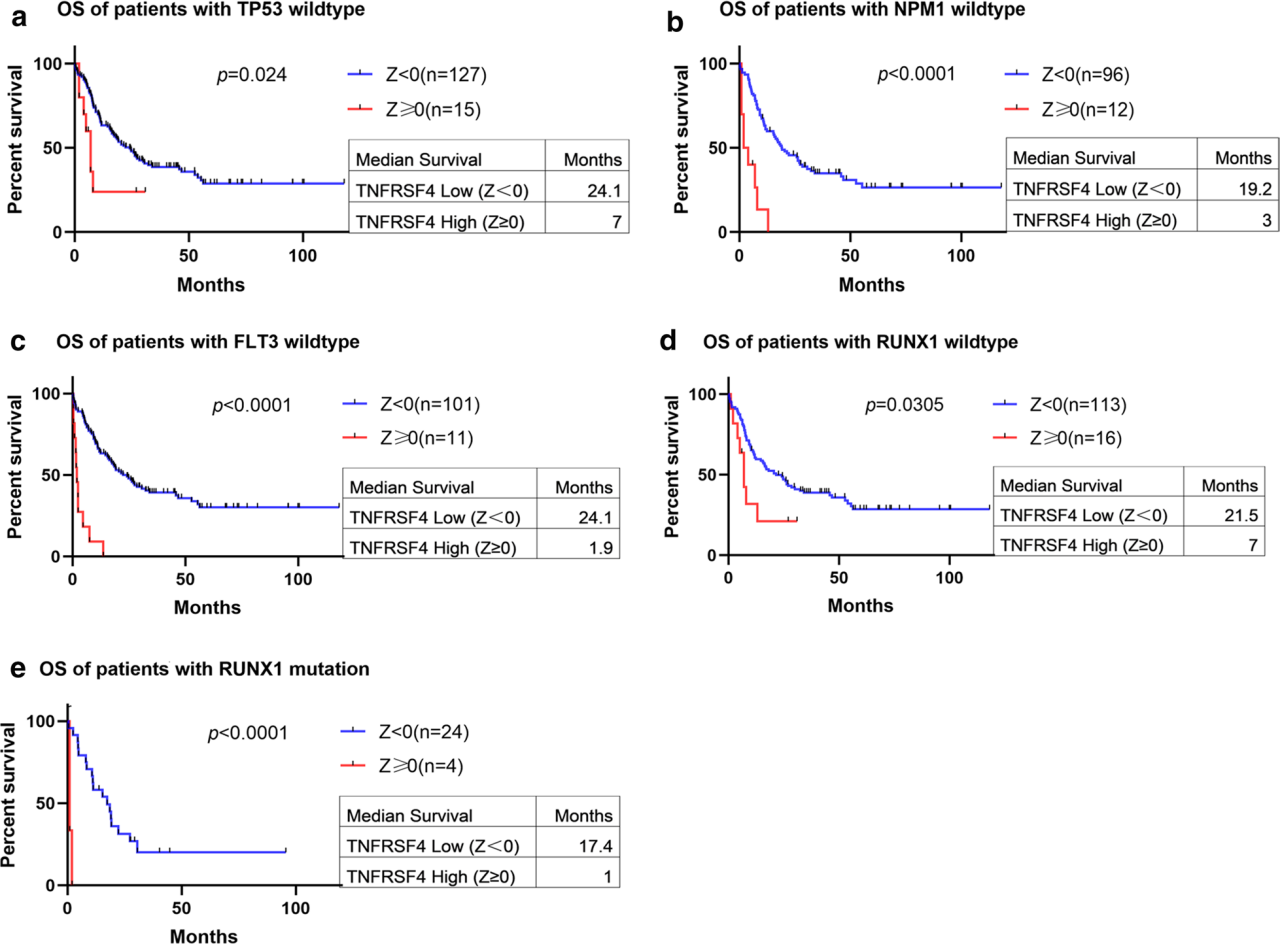
Survival analysis of patients with respect to *TNFRSF4* expression after stratification based on *TP53*, *NPM1*, *FLT3* and *RUNX1* mutation status. Overall survival of patients with *TNFRSF4* high (Z score ≥ 0) versus *TNFRSF4* low (Z score < 0) among patients with **a***TP53*, **b***NPM1*, **c***FLT3* and **d***RUNX1* wild-type gene. **e** Overall survival of patients with *TNFRSF4* high (Z score ≥ 0) versus *TNFRSF4* low (Z score < 0) among patients with *RUNX1* mutated gene

Additionally, we examined the association between *TNFRSF4* expression and clinical outcome in patients with *TP53*, *NPM1*, *FLT3* and *RUNX1* mutations. We stratified patients according to mutational status and performed survival analysis in each group. We found that in patients with wild type *TP53*, *NPM1*, *FLT3* and *RUNX1*, high *TNFRSF4* expression (Z ≥ 0) was associated with a significantly shorter OS (median survival: *TP53*, 7 months vs. 24.1 months, *p *= 0.024; *NPM1*, 3 months vs. 19.2 months, *p *< 0.0001; *FLT3*, 1.9 months vs. 24.1 months, *p *< 0.0001; *RUNX1*, 7 months vs. 21.5 months, *p *= 0.0305 Fig. 4a–d). There was a similar trend but not statistically significant association was observed with high *TNFRSF4* expression and shorter DFS (median survival: *TP53*, 15 months vs. 16.1 months, *p *= 0.0507; *NPM1*, 12 months vs. 17 months, *p *= 0.0661; *FLT3*, 12 months vs. 17.3 months, *p *= 0.6943; *RUNX1*, 12 months vs. 14.2 months, *p *= 0.0650, Additional file 3: Figure S2A–D). We also found that only in patients with *RUNX1* mutation, high *TNFRSF4* expression was associated with a significantly shorter OS (median survival: 1 months vs. 17.4 months, *p *< 0.0001, Fig. 4e).

### *TNFRSF4* expression and clinical outcome

**Fig. 5:**
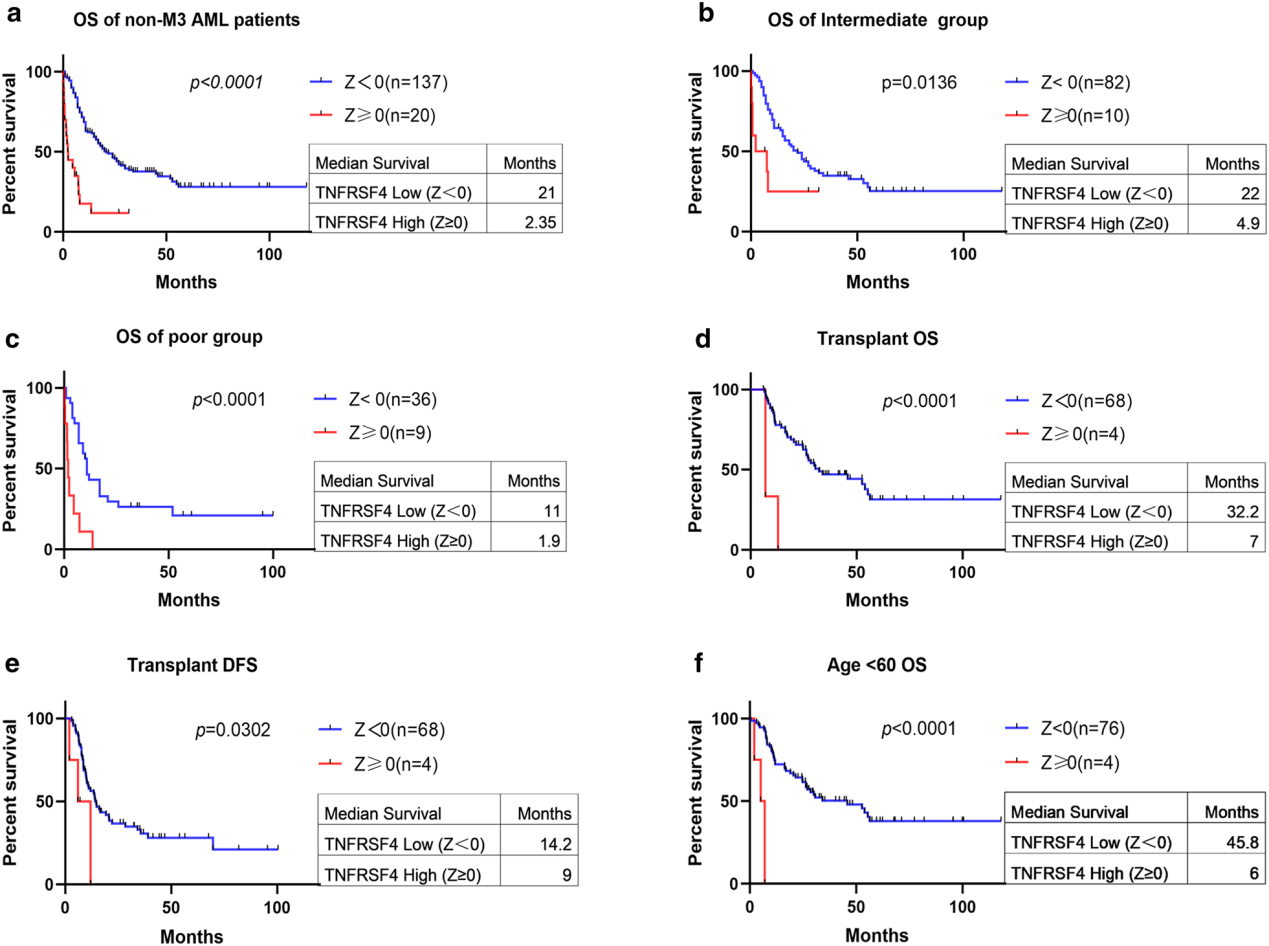
Survival analysis of patients with respect to *TNFRSF4* expression. **a** Overall survival of 157 non-M3 AML patients with *TNFRSF4* Z score ≥ 0 and *TNFRSF4* Z score < 0. Survival analysis of patients with respect to *TNFRSF4* expression based on patient risk stratification. **b** Overall survival of patients with *TNFRSF4* high (Z score ≥ 0) versus *TNFRSF4* low (Z score < 0) in patients with intermediate risk stratification. **c** Overall survival of patients with *TNFRSF4* high (Z score ≥ 0) versus *TNFRSF4* low (Z score < 0) in patients with poor risk stratification. Survival analysis of patients with respect to *TNFRSF4* expression after stratification based on patient transplant status. **d** Overall survival and **e** disease-free survival of patients with *TNFRSF4* high (Z score ≥ 0) versus *TNFRSF4* low (Z score < 0) in patients who received a transplant. Survival analysis of patients with respect to *TNFRSF4* expression based on age. **f** Overall survival of patients < 60 years of age with *TNFRSF4* high (Z score ≥ 0) versus *TNFRSF4* low (Z score < 0)

The OS of *TNFRSF4* high group (Z ≥ 0) was significantly shorter than that of low expression patients (median survival: 2.35 months vs. 21 months, *p *< 0.0001, Fig. 5a), A similar trend was observed in DFS in patients with high *TNFRSF4* expression (median survival: 12 months vs. 14.6 months, *p* = 0.0868; Additional file 3: Figure S2E). To further validate the association between high *TNFRSF4* and poor clinical outcome, we stratified patients into Z ≥ 0.5 and Z < 0.5 for survival analysis. Patients with high *TNFRSF4* (Z ≥ 0.5) expression had significantly shorter OS than patients with low *TNFRSF4* (median survival: 0.8 months vs. 19 months, *p *< 0.0001; Additional file 3: Figure S2F). We also analyzed the TCGA data set using *TNFRSF4* median expression to dichotomize patients into high and low expression groups. We found that patients with high *TNFRSF4* expression had shorter OS (median survival: months 11.5 vs. 22 months, *p* = 0.0235; Additional file 3: Figure S2G).

Non-M3 patients were also grouped according to FAB classification system. Among M0, M1, M2 and M4 subtypes, patients with high *TNFRSF4* (Z ≥ 0) had significantly shorter OS (median survival: M0, 2.4 months vs. 26 months, *p *< 0.0001; M1, 4 months vs. 27 months, *p *= 0.0163; M2, 0.8 months vs. 19 months, *p *= 0.004; M4, 4.85 months vs. 19 months, *p *= 0.0101; Additional file 4: Figure S3A–D). Furthermore, there was a significant decrease in the DFS of *TNFRSF4* high patients (Z ≥ 0) compared with *TNFRSF4* low patients (median survival: months 5.15 vs. 13 months, *p* = 0.004; Additional file 4: Figure S3E) in M1 subgroup but not in the rest subgroups.

When patients were stratified according to their risk stratification, we found that among intermediated and poor risk stratification patients, those *TNFRSF4* high (Z ≥ 0) had shorter OS than those with *TNFRSF4* low (median survival: intermediated, months 4.9 vs. 22 months, *p *= 0.0136; Poor, 1.9 vs. 11 months, *p *< 0.0001; Fig. 5b, c). A similar but no statistically significant trend of DFS was observed in poor risk stratification (median survival: 9 months vs. 17 months, *p *= 0.3376; Additional file 5: Figure S4A). However, no significant difference was observed in DFS between *TNFRSF4* high and low group in intermediated risk stratification.

When patients were stratified according to whether they received a transplant or not, we found that only in patients who received a transplant with *TNFRSF4* high expression (Z ≥ 0) associated with significantly shorter OS and DFS (median OS: 7 months vs. 32.3 months, *p *< 0.0001; median DFS: 9 months vs. 14.2 months, *p *= 0.0302; Fig. 5d, e). No significant difference but similar trend was observed in OS and DFS between the *TNFRSF4* high and low in patients who did not receive a transplant (median OS: 3 months vs. 9.55 months, *p *= 0.1503; median DFS: 15 months vs. 17 months, *p *= 0.4539; Additional file 5: Figure S4B, C).

We performed survival analysis in patients with non-M3 AML stratified by their age into younger patients (< 60) and older patients (≥ 60). In patients < 60, high *TNFRSF4* (Z ≥ 0) expression was associated with shorter OS compared with low *TNFRSF4* (median survival: 6 months vs. 45.8 months, *p *< 0.0001; Fig. 5f) and a decrease in DFS though not significant (median survival: 6.5 months vs. 16.1 months, *p *= 0.2047, Additional file 5: Figure S4D). A similar trend but not statistically significant association of OS and DFS was observed in older patients (median OS: 6 months vs. 10.5 months; *p *= 0.6007; median DFS: 12 months vs. 13.4 months; *p *= 0.1207; Additional file 5: Figure S4E, F).

## Discussion

In present study, we analyzed DEGs between *TP53*-mutated and wildtype non-M3 AML patients based on TCGA dataset. IL15 signaling pathway as well as cytokine–cytokine receptor interaction pathway and immune response pathway were screened out after enrichment analysis. We also utilized the “String” website to complete the PPI networks of DEGs from each pathway and imported PPI networks into Cytoscape plug-ins to found out the candidate genes. Subsequently, *TNFRSF4* was screened out by overlapping candidate genes and was used for further study.

Here we reported that elevated *TNFRSF4* mRNA expression is significantly associated with poorer OS of non-M3 AML patients. This is particularly relevant with patients < 60 years of age, patients received transplant and patients in intermediate and poor risk stratification. The significant shorter OS of high *TNFRSF4* mRNA expression patients is also relevant with *TP53*, *NPM1*, *FLT3* and *RUNX1* wild type. Additionally, combined with our clinical BM simples, *TNFRSF4* mRNA expression was higher in AML patients than HDs and MDS (EB-1, 2) patients and the expression is positively related with BM blasts percentage. Therefore, the identification of outcome predictors and possible viable targets in aforementioned subset of patients will greatly affect disease understanding and treatment outcome.

Cancer immunotherapy is emerging as a promising approach for cancer treatment and immune checkpoint inhibitors have advanced rapidly over the past decade. Anti-cytotoxic T-lymphocyte antigen 4 (CTLA4) and anti-programmed death 1 (PD1)/Programmed death-ligand 1 (PD-L1) monoclonal antibodies have produced long-lasting anti-tumor immune responses that translate into clinical benefits for many cancer types [29]. Previous research has found that p53 can transactivate a number of immunosuppressive genes including programmed death–ligand 1 (PD-L1), thus activating one of the major immunological checkpoints. p53 also transactivates the expression of forkhead box P3 (FOXP3), a transcription factor that is essential for the generation and function of regulatory T cells, a subpopulation that maintains tolerance to self-antigens [30]. Breast cancers with *TP53* mutation also show significantly higher activities of a wide variety of immune cells, functions, and pathways than *TP53* wildtype group [18]. In AML, *TP53* mutation is associated with complex karyotype, poor standard therapy response and short overall survival [10, 11]. However, the role of *TP53* in AML is enigmatic. In our research, *TNFRSF4* was screened out by dividing patients based on absence or presence of *TP53* mutation, thereby we speculated that the mechanism between *TNFRSF4* and *TP53* maybe associated with IL15 related immune response or cytokine–cytokine receptor interaction. We found that beside *TP53*, *NPM1* and *FLT3* mutation also associated with high *TNFRSF4* expression. The mechanistic interplay between *TNFRSF4* and the mutation genes is yet to be determined.

TNFRSF4/TNFSF4 signaling serves a key role in the development, differentiation and physiological functions of T cells and other immunological cells [31]. In various tumor models, anti-TNFRSF4 has been shown to enhance CD8+ T cells infiltration and reduce Treg cells infiltration into the tumor [17, 32, 33]. Another research also found a dependence on direct TNFRSF4 ligation on CD8+ T cells to increase tumor specific cytotoxicity in vivo [16]. Research has found that AML patients CD8+ T cell dysfunction was in part reversible upon PD-1 blockade or TNFRSF4 co-stimulation in vitro [34]. Besides T cells, NK cells are a second cytotoxic lymphocyte subset that contributes to antitumor immunity, particularly in leukemia [35]. Research reported that TNFRSF4 is expressed on AML blasts, depending on TNFRSF4/TNFSF4 signaling promoted NK-cell activation, cytokine production and cytotoxicity which can against primary AML cells [36]. In combination with our results, we suggested that immunotherapy may product more therapeutic effect in patients with high *TNFRSF4* expression.

## Conclusion

*TNFRSF4* was screened out as a key gene related with *TP53* mutation based on non-M3 AML TCGA data set. *TNFRSF4* was higher in intermediate, poor risk stratification and related with relapse status. Additionally, high *TNFRSF4* expression was also associated with *NPM1*, *FLT3* mutation. Based our clinical data, we found *TNFRSF4* expression was significant higher in non-M3 AML patients than HDs and MDS (EB-1, 2) patients. The expression level was positively related with blasts percentage. Our findings demonstrate that elevated *TNFRSF4* expression contributes to predict the poor clinical outcome of patients with non-M3 AML. This study provides a rationale for further functional and mechanistic studies aiming to understand the role of *TNFRSF4* in non-M3 AML.

## Supplementary information

**Additional file 1: Table S1.** The statistical methods used in present research. **Table S2.** The top 15 genes with the highest score of each pathway through the Cytoscape “cytoHubba” module analysis.

**Additional file 2: Figure S1.** (A) Wayne diagram with 5 overlapping DEGs from cytokine–cytokine receptor interaction pathway, immune response pathway and the IL15 signaling pathway. (B) The box plot from GEPIA2 matched TCGA normal and GTEx AML data with log2(TPM + 1) for log-scale. *p < 0.05. *TNFRSF4* Z-score and mRNA expression distribution. (C) Histogram of *TNFRSF4* Log2 transformed mRNA expression; (D) *TNFRSF4* mRNA expression Z score; (E) Scatterplot of mRNA Z-score vs mRNA log2 mRNA expression. Relative *TNFRSF4* log2 mRNA expression categorized by (F) FAB classification and (G) NCCN risk stratification. (H) *TNFRSF4* expression in relapsed vs. de novo non-M3 AML samples from the BEAT AML. **p < 0.01, ***p < 0.001.

**Additional file 3: Figure S2.** Survival analysis of patients with respect to *TNFRSF4* expression after stratification based on *TP53*, *NPM1*, *FLT3* and *RUNX1* mutation status. Disease-free survival of patients with *TNFRSF4* high (Z score ≥ 0) versus *TNFRSF4* low (Z score < 0) among patients with (A)*TP53*, (B) *NPM1*, (C) *FLT3* and (D) *RUNX1* wild-type gene. Survival analysis of patients with respect to *TNFRSF4* expression. (E) Disease-free survival of patients with *TNFRSF4* Z score ≥ 0 and *TNFRSF4* Z score < 0. (F) Overall survival of patients with *TNFRSF4* Z score ≥ 0.5 and *TNFRSF4* Z score < 0.5. (G) Overall survival of patients that dichotomized based on *TNFRSF4* median mRNA expression into *TNFRSF4* high and *TNFRSF4* low according to the log2 median-centered expression.

**Additional file 4: Figure S3.** Survival analysis of patients with respect to *TNFRSF4* expression after stratification based on FAB classification. Overall survival of patients with *TNFRSF4* high (Z score ≥ 0) versus *TNFRSF4* low (Z score < 0) among patients with (A) M0, (B) M1, (C) M2 and (D) M4 classification. (E) Disease-free survival of patients with *TNFRSF4* high (Z score ≥ 0) versus *TNFRSF4* low (Z score < 0) among patients with M1 classification.

**Additional file 5: Figure S4.** Survival analysis of patients with respect to *TNFRSF4* expression based on patient risk stratification. (A) Disease-free survival of patients with *TNFRSF4* high (Z score ≥ 0) versus *TNFRSF4* low (Z score < 0) in patients with poor risk stratification. Survival analysis of patients with respect to *TNFRSF4* expression after stratification based on patient transplant status. (B) Overall survival and (C) disease-free survival of patients with *TNFRSF4* high (Z score ≥ 0) versus *TNFRSF4* low (Z score < 0) in patients who did not received a transplant. Survival analysis of patients with respect to *TNFRSF4* expression based on age. (D) Disease-free survival of patients < 60 years of age with *TNFRSF4* high (Z score ≥ 0) versus *TNFRSF4* low (Z score < 0). (E) Overall survival and (F) disease-free survival of patients ≥ 60 years of age with *TNFRSF4* high (Z score ≥ 0) versus *TNFRSF4* low (Z score < 0).

## Data Availability

The 200 AML patient datasets obtained from the Cancer Genome Atlas (TCGA) at cBioPortal (https://www.cbioportal.org). The clinical patient datasets for the current study are not publicly accessible in accordance with local health research ethics protocols; however, it may be available from the corresponding author.

## Abbreviations

AML: Acute myeloid leukemia
TNFRSF4: Tumor necrosis factor receptor superfamily member 4
CD8+: CD8-positive
HDs: Healthy donors
MDS: Myelodysplastic syndrome
EB: Excess blasts
DEGs: Differentially expressed genes
TCGA: The Cancer Genome Atlas
FAB: French–American–British
NCCN: National Comprehensive Cancer Network
log2FC: log2FoldChange
KEGG: The Kyoto Encyclopedia of Genes and Genome
GO: Gene Ontology
DAVID: Database for Annotation, Visualization and Integrated Discovery
FDR: False discovery rate
GSEA: Gene set enrichment analysis
MSigDB: Molecular Signatures Database
MCC: Maximal Clique Centrality
RT-qPCR: Reverse transcribed quantitative PCR
BMCs: Bone marrow cells
OS: Overall survival
DFS: Disease free survival
PPI: Protein–protein interaction
CTLA4: Anti-cytotoxic T-lymphocyte antigen 4
PD1: Anti-programmed death 1
PD-L1: Programmed death-ligand 1
FOXP3: Forkhead box P3

## References

[1] Dohner H, Weisdorf DJ, Bloomfield CD (2015). Acute myeloid leukemia. N Engl J Med.

[2] Arber DA, Orazi A, Hasserjian R, Thiele J, Borowitz MJ, Le Beau MM, Bloomfield CD, Cazzola M, Vardiman JW (2016). The 2016 revision to the World Health Organization classification of myeloid neoplasms and acute leukemia. Blood.

[3] Ley TJ, Miller C, Ding L, Raphael BJ, Mungall AJ, Robertson A, Hoadley K, Triche TJ, Laird PW, Baty JD (2013). Genomic and epigenomic landscapes of adult de novo acute myeloid leukemia. N Engl J Med.

[4] Papaemmanuil E, Gerstung M, Bullinger L, Gaidzik VI, Paschka P, Roberts ND, Potter NE, Heuser M, Thol F, Bolli N (2016). Genomic classification and prognosis in acute myeloid leukemia. N Engl J Med.

[5] Lane DP (1992). Cancer p53, guardian of the genome. Nature.

[6] Williams AB, Schumacher B (2016). p53 in the DNA-damage-repair process. Cold Spring Harb Perspect Med.

[7] Oren M, Rotter V (1999). Introduction: p53–the first twenty years. Cell Mol Life Sci.

[8] Kadia TM, Jain P, Ravandi F, Garcia-Manero G, Andreef M, Takahashi K, Borthakur G, Jabbour E, Konopleva M, Daver NG (2016). TP53 mutations in newly diagnosed acute myeloid leukemia: clinicomolecular characteristics, response to therapy, and outcomes. Cancer.

[9] Ok CY, Patel KP, Garcia-Manero G, Routbort MJ, Peng J, Tang G, Goswami M, Young KH, Singh R, Medeiros LJ (2015). TP53 mutation characteristics in therapy-related myelodysplastic syndromes and acute myeloid leukemia is similar to de novo diseases. J Hematol Oncol.

[10] Schoch C, Kern W, Kohlmann A, Hiddemann W, Schnittger S, Haferlach T (2005). Acute myeloid leukemia with a complex aberrant karyotype is a distinct biological entity characterized by genomic imbalances and a specific gene expression profile. Genes Chromosomes Cancer.

[11] Rucker FG, Schlenk RF, Bullinger L, Kayser S, Teleanu V, Kett H, Habdank M, Kugler CM, Holzmann K, Gaidzik VI (2012). TP53 alterations in acute myeloid leukemia with complex karyotype correlate with specific copy number alterations, monosomal karyotype, and dismal outcome. Blood.

[12] Croft M, So T, Duan W, Soroosh P (2009). The significance of OX40 and OX40L to T-cell biology and immune disease. Immunol Rev.

[13] Kawamata S, Hori T, Imura A, Takaori-Kondo A, Uchiyama T (1998). Activation of OX40 signal transduction pathways leads to tumor necrosis factor receptor-associated factor (TRAF) 2- and TRAF5-mediated NF-kappaB activation. J Biol Chem.

[14] Watts TH (2005). TNF/TNFR family members in costimulation of T cell responses. Annu Rev Immunol.

[15] Linch SN, McNamara MJ, Redmond WL (2015). OX40 agonists and combination immunotherapy: putting the pedal to the metal. Front Oncol.

[16] Gough MJ, Crittenden MR, Sarff M, Pang P, Seung SK, Vetto JT, Hu HM, Redmond WL, Holland J, Weinberg AD (2010). Adjuvant therapy with agonistic antibodies to CD134 (OX40) increases local control after surgical or radiation therapy of cancer in mice. J Immunother.

[17] Aspeslagh S, Postel-Vinay S, Rusakiewicz S, Soria JC, Zitvogel L, Marabelle A (2016). Rationale for anti-OX40 cancer immunotherapy. Eur J Cancer.

[18] Liu Z, Jiang Z, Gao Y, Wang L, Chen C, Wang X (2019). TP53 mutations promote immunogenic activity in breast cancer. J Oncol.

[19] Williams P, Basu S, Garcia-Manero G, Hourigan CS, Oetjen KA, Cortes JE, Ravandi F, Jabbour EJ, Al-Hamal Z, Konopleva M (2019). The distribution of T-cell subsets and the expression of immune checkpoint receptors and ligands in patients with newly diagnosed and relapsed acute myeloid leukemia. Cancer.

[20] Cerami E, Gao J, Dogrusoz U, Gross BE, Sumer SO, Aksoy BA, Jacobsen A, Byrne CJ, Heuer ML, Larsson E (2012). The cBio cancer genomics portal: an open platform for exploring multidimensional cancer genomics data. Cancer Discov.

[21] Pashaiefar H, Yaghmaie M, Tavakkoly-Bazzaz J, Ghaffari SH, Alimoghaddam K, Momeny M, Izadi P, Izadifard M, Kasaeian A, Ghavamzadeh A (2018). PARP-1 overexpression as an independent prognostic factor in adult non-M3 acute myeloid leukemia. Genet Test Mol Biomarkers.

[22] Love MI, Huber W, Anders S (2014). Moderated estimation of fold change and dispersion for RNA-seq data with DESeq2. Genome Biol.

[23] Ito K, Murphy D (2013). Application of ggplot2 to pharmacometric graphics. CPT Pharmacomet Syst Pharmacol.

[24] da Huang W, Sherman BT, Lempicki RA (2009). Systematic and integrative analysis of large gene lists using DAVID bioinformatics resources. Nat Protoc.

[25] Subramanian A, Tamayo P, Mootha VK, Mukherjee S, Ebert BL, Gillette MA, Paulovich A, Pomeroy SL, Golub TR, Lander ES (2005). Gene set enrichment analysis: a knowledge-based approach for interpreting genome-wide expression profiles. Proc Natl Acad Sci.

[26] Szklarczyk D, Gable AL, Lyon D, Junge A, Wyder S, Huerta-Cepas J, Simonovic M, Doncheva NT, Morris JH, Bork P (2019). STRING v11: protein–protein association networks with increased coverage, supporting functional discovery in genome-wide experimental datasets. Nucleic Acids Res.

[27] Shannon P, Markiel A, Ozier O, Baliga NS, Wang JT, Ramage D, Amin N, Schwikowski B, Ideker T (2003). Cytoscape: a software environment for integrated models of biomolecular interaction networks. Genome Res.

[28] Tyner JW, Tognon CE, Bottomly D, Wilmot B, Kurtz SE, Savage SL, Long N, Schultz AR, Traer E, Abel M (2018). Functional genomic landscape of acute myeloid leukaemia. Nature.

[29] Couzin-Frankel J (2013). Breakthrough of the year 2013. Cancer immunotherapy. Science.

[30] Zitvogel L, Kroemer G (2015). A p53-regulated immune checkpoint relevant to cancer. Science.

[31] Kow NY, Mak A (2013). Costimulatory pathways: physiology and potential therapeutic manipulation in systemic lupus erythematosus. Clin Dev Immunol.

[32] Gough MJ, Ruby CE, Redmond WL, Dhungel B, Brown A, Weinberg AD (2008). OX40 agonist therapy enhances CD8 infiltration and decreases immune suppression in the tumor. Cancer Res.

[33] Pardee AD, McCurry D, Alber S, Hu P, Epstein AL, Storkus WJ (2010). A therapeutic OX40 agonist dynamically alters dendritic, endothelial, and T cell subsets within the established tumor microenvironment. Cancer Res.

[34] Knaus HA, Berglund S, Hackl H, Blackford AL, Zeidner JF, Montiel-Esparza R, Mukhopadhyay R, Vanura K, Blazar BR, Karp JE (2018). Signatures of CD8+ T cell dysfunction in AML patients and their reversibility with response to chemotherapy. JCI Insight.

[35] Pierson BA, Miller JS (1996). CD56 + bright and CD56 + dim natural killer cells in patients with chronic myelogenous leukemia progressively decrease in number, respond less to stimuli that recruit clonogenic natural killer cells, and exhibit decreased proliferation on a per cell basis. Blood.

[36] Nuebling T, Schumacher CE, Hofmann M, Hagelstein I, Schmiedel BJ, Maurer S, Federmann B, Rothfelder K, Roerden M, Dorfel D (2018). The immune checkpoint modulator OX40 and its ligand OX40L in NK-cell immunosurveillance and acute myeloid leukemia. Cancer Immunol Res.

