# Supplementary material for: Elevated TNFRSF4 gene expression is a predictor of poor prognosis in non-M3 acute myeloid leukemia
Source: Cancer Cell Int. 2020 May 4;20:146. doi: 10.1186/s12935-020-01213-y (PMC7197135; doi:10.1186/s12935-020-01213-y)
Supplement: Supplementary file 1 — Additional file 1: Table S1. The statistical methods used in present research. Table S2. The top 15 genes with the highest score of each pathway through the Cytoscape “cytoHubba” module analysis. [file 12935_2020_1213_MOESM1_ESM.docx]

Table S1. The statistical methods used in present research.

| **statistic methods** | **analysis** |
| --- | --- |
| Log-rank (Mantel-Cox) test | Survival analysis. |
| Mann−Whitney U’s nonparametric t test | Compare TNFRSF4 expression level between groups. |
| Fisher’s exact test | Compare the distribution of high and low TNFRSF4 expression patients in different gender and different risk stratification. |
| Pearson correlation coefficient | Analysis the correlation between TNFRSF4 log2-transformed mRNA expression and TNFRSF4 Z score. |
| Spearman correlation coefficient | Analysis the correlation between TNFRSF4 expression level and bone marrow blasts based on our clinical data. |

Table S2: The top 15 genes with the highest score of each pathway through the Cytoscape “cytoHubba” module analysis.

| **Top 15** | **IL15_UP. V1_UP pathway** | | **Cytokine-cytokine receptor interaction pathway** | | **Immune response pathway** | |
| --- | --- | --- | --- | --- | --- | --- |
| **Rank** | **Name** | **Score** | **Name** | **Score** | **Name** | **Score** |
| 1 | IL10 | 449 | CCR7 | 4.46E+06 | CD86 | 1.13E+09 |
| 2 | FOXP3 | 418 | CD40LG | 4.46E+06 | CCR7 | 1.13E+09 |
| 3 | CCL4 | 402 | IL7 | 4.46E+06 | CD40LG | 1.13E+09 |
| 4 | CD69 | 384 | TNFRSF18 | 4.44E+06 | CTLA4 | 1.13E+09 |
| 5 | CXCL12 | 251 | FASLG | 4.40E+06 | IL7 | 1.13E+09 |
| 6 | SLAMF1 | 249 | CD70 | 4.40E+06 | CD274 | 1.13E+09 |
| 7 | TNFRSF4 | 144 | TNFRSF9 | 4.39E+06 | TNFRSF18 | 1.12E+09 |
| 8 | CCL21 | 132 | IL2RB | 4.05E+06 | TNFRSF9 | 1.08E+09 |
| 9 | BYSL | 126 | TNFRSF4 | 4.04E+06 | CD27 | 1.04E+09 |
| 10 | NOB1 | 122 | CD27 | 4.04E+06 | FASLG | 1.04E+09 |
| 10 | TSR1 | 122 | IL7R | 4.04E+06 | TNFRSF4 | 1.04E+09 |
| 12 | RIOK2 | 120 | CD40 | 4.12E+05 | CD70 | 1.04E+09 |
| 12 | PNO1 | 120 | TNFSF9 | 4.03E+05 | IL7R | 6.11E+08 |
| 12 | RPP25 | 120 | CXCR5 | 5.54E+04 | TNFSF9 | 5.19E+08 |
| 15 | FOS | 59 | CCL4 | 1.56E+04 | ICOS | 8.78E+07 |
