# Supplementary figures and images for: Elevated TNFRSF4 gene expression is a predictor of poor prognosis in non-M3 acute myeloid leukemia
Source: Cancer Cell Int. 2020 May 4;20:146. doi: 10.1186/s12935-020-01213-y (PMC7197135; doi:10.1186/s12935-020-01213-y)

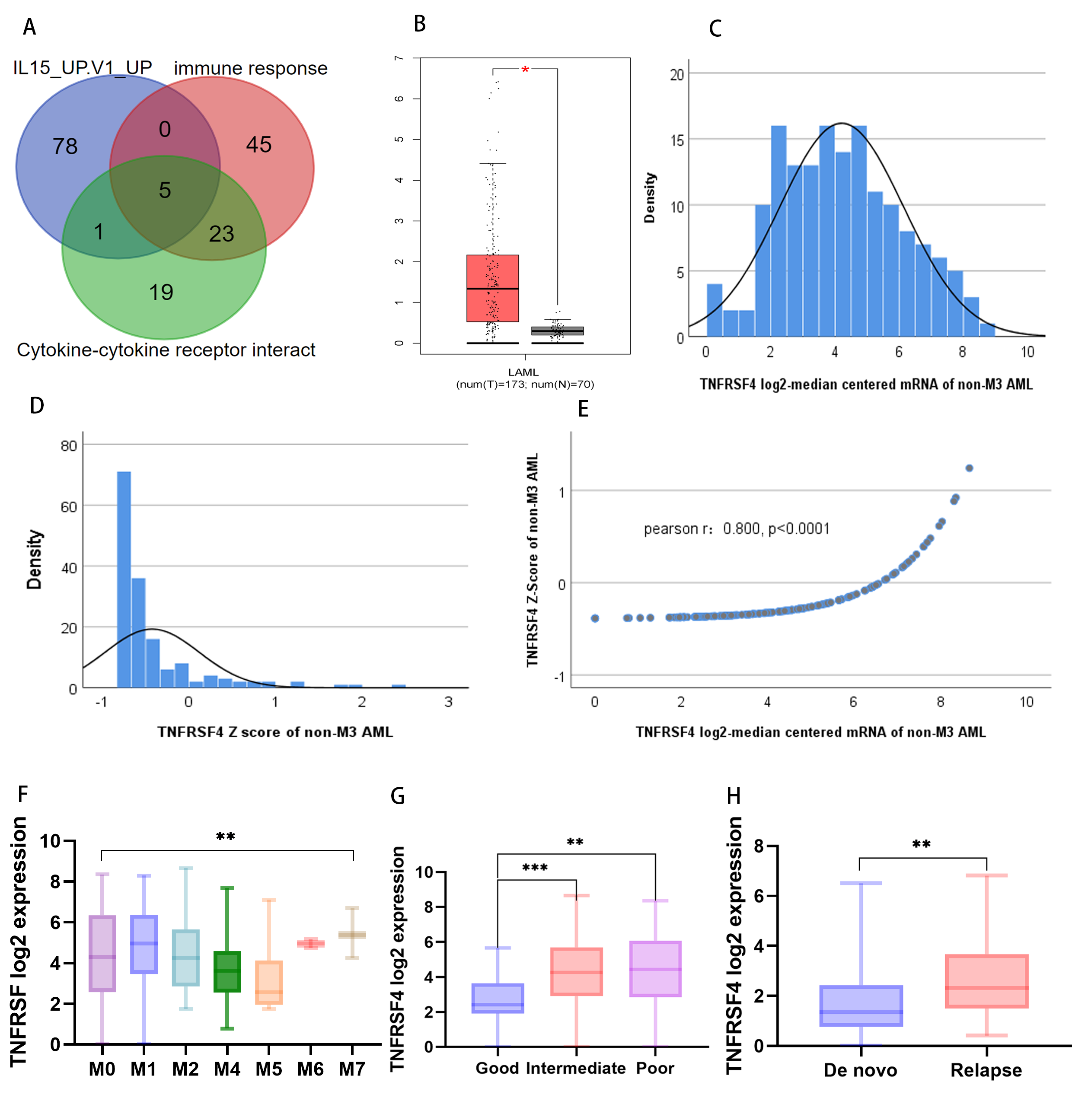

Supplement: Supplementary file 2 — Additional file 2: Figure S1. (A) Wayne diagram with 5 overlapping DEGs from cytokine–cytokine receptor interaction pathway, immune response pathway and the IL15 signaling pathway. (B) The box plot from GEPIA2 matched TCGA normal and GTEx AML data with log2(TPM + 1) for log-scale. *p < 0.05. TNFRSF4 Z-score and mRNA expression distribution. (C) Histogram of TNFRSF4 Log2 transformed mRNA expression; (D) TNFRSF4 mRNA expression Z score; (E) Scatterplot of mRNA Z-score vs mRNA log2 mRNA expression. Relative TNFRSF4 log2 mRNA expression categorized by (F) FAB classification and (G) NCCN risk stratification. (H) TNFRSF4 expression in relapsed vs. de novo non-M3 AML samples from the BEAT AML. **p < 0.01, ***p < 0.001. [file 12935_2020_1213_MOESM2_ESM.png]

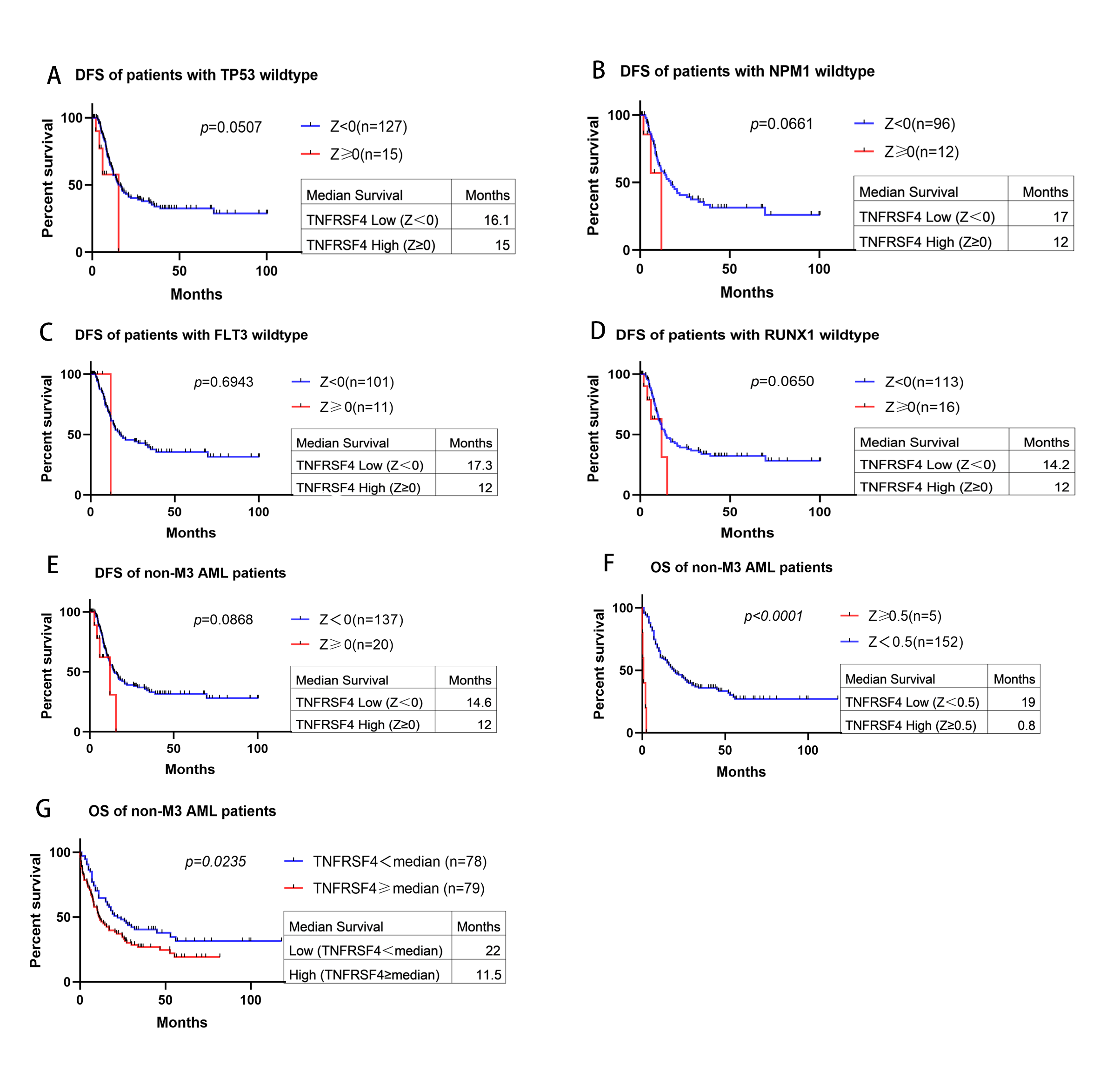

Supplement: Supplementary file 3 — Additional file 3: Figure S2. Survival analysis of patients with respect to TNFRSF4 expression after stratification based on TP53, NPM1, FLT3 and RUNX1 mutation status. Disease-free survival of patients with TNFRSF4 high (Z score ≥ 0) versus TNFRSF4 low (Z score < 0) among patients with (A)TP53, (B) NPM1, (C) FLT3 and (D) RUNX1 wild-type gene. Survival analysis of patients with respect to TNFRSF4 expression. (E) Disease-free survival of patients with TNFRSF4 Z score ≥ 0 and TNFRSF4 Z score < 0. (F) Overall survival of patients with TNFRSF4 Z score ≥ 0.5 and TNFRSF4 Z score < 0.5. (G) Overall survival of patients that dichotomized based on TNFRSF4 median mRNA expression into TNFRSF4 high and TNFRSF4 low according to the log2 median-centered expression. [file 12935_2020_1213_MOESM3_ESM.png]

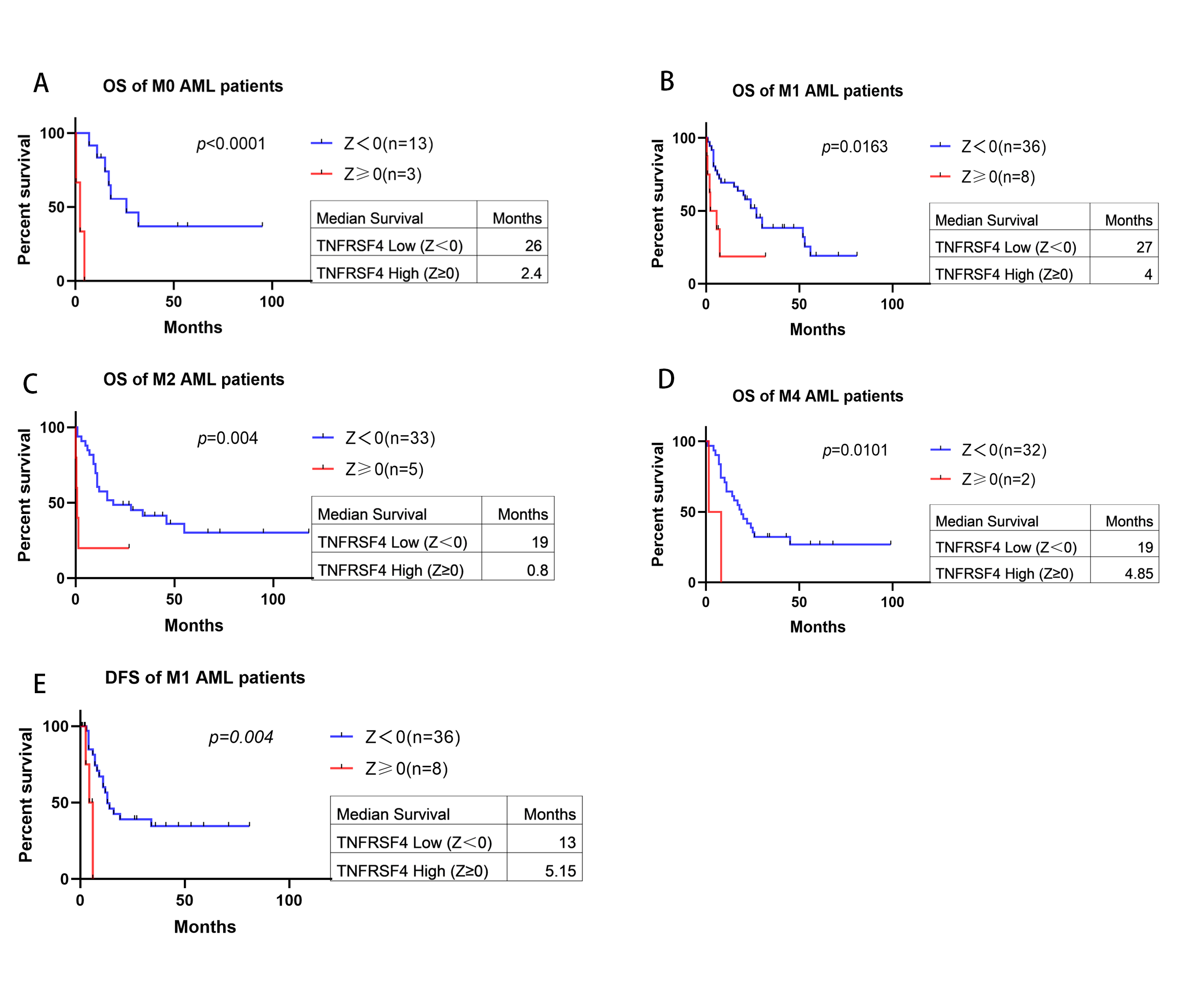

Supplement: Supplementary file 4 — Additional file 4: Figure S3. Survival analysis of patients with respect to TNFRSF4 expression after stratification based on FAB classification. Overall survival of patients with TNFRSF4 high (Z score ≥ 0) versus TNFRSF4 low (Z score < 0) among patients with (A) M0, (B) M1, (C) M2 and (D) M4 classification. (E) Disease-free survival of patients with TNFRSF4 high (Z score ≥ 0) versus TNFRSF4 low (Z score < 0) among patients with M1 classification. [file 12935_2020_1213_MOESM4_ESM.png]

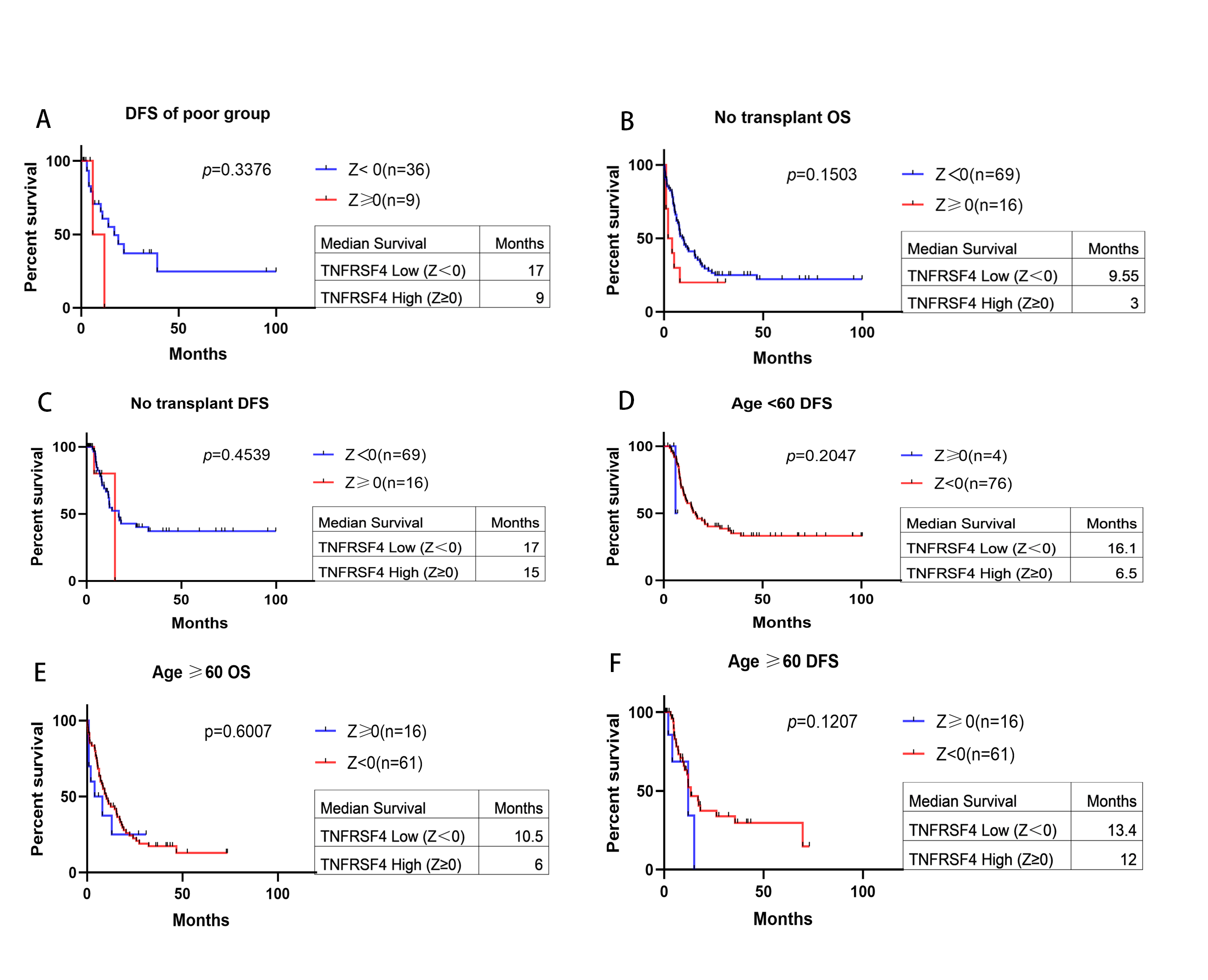

Supplement: Supplementary file 5 — Additional file 5: Figure S4. Survival analysis of patients with respect to TNFRSF4 expression based on patient risk stratification. (A) Disease-free survival of patients with TNFRSF4 high (Z score ≥ 0) versus TNFRSF4 low (Z score < 0) in patients with poor risk stratification. Survival analysis of patients with respect to TNFRSF4 expression after stratification based on patient transplant status. (B) Overall survival and (C) disease-free survival of patients with TNFRSF4 high (Z score ≥ 0) versus TNFRSF4 low (Z score < 0) in patients who did not received a transplant. Survival analysis of patients with respect to TNFRSF4 expression based on age. (D) Disease-free survival of patients < 60 years of age with TNFRSF4 high (Z score ≥ 0) versus TNFRSF4 low (Z score < 0). (E) Overall survival and (F) disease-free survival of patients ≥ 60 years of age with TNFRSF4 high (Z score ≥ 0) versus TNFRSF4 low (Z score < 0). [file 12935_2020_1213_MOESM5_ESM.png]
